# Gingival recession: prevalence and risk indicators among young greek adults

**DOI:** 10.4317/jced.51354

**Published:** 2014-07-01

**Authors:** Nikolaos A. Chrysanthakopoulos

**Affiliations:** 1 Dental Surgeon D.D.Sc. Resident in Maxillofacial and Oral Surgery, 401 General Military Hospital of Athens, Athens, Greece; 2 Post-Graduate Student in Dept. of Pathological Anatomy, Medical School, University of Athens, Athens, Greece

## Abstract

Objectives: The aim of the current research was to assess the prevalence of gingival recession and to investigate possible associations among this condition, periodontal and epidemiological variables in a sample of young Greek adults in a general dental practice. Material and Methods: A total of 1,430 young adults was examined clinically and interviewed regarding several periodontal and epidemiological variables. Collected data included demographic variables, oral hygiene habits and smoking status. Clinical examination included the recording of dental plaque, supragingival calculus presence, gingival status and buccal gingival recession. Multivariate logistic regression analysis model was performed to access the possible association between gingival recession and several periodontal and epidemiological variables as potential risk factors. Results: The overall prevalence of gingival recession was 63.9%. The statistical analysis indicated that higher educational level [OR= 2.12, 95% CI= 0.53-8.51], cigarette smoking [OR= 1.97, 95% CI= 1.48-7.91], frequent tooth brushing [OR= 0.98, 95% CI= 0.56-1.96], presence of oral piercing [OR= 0.92, 95% CI= 0.38-1.58], presence of gingival inflammation [OR= 4.54, 95% CI= 1.68-7.16], presence of dental plaque [OR= 1.67, 95% CI= 0.68-2.83] and presence of supragingival calculus [OR=1.34, 95% CI= 0.59-1.88], were the most important associated factors of gingival recession. Conclusions: The observations of the current research supported the results from previous authors that several periodontal factors, educational level and smoking were significantly associated with the presence of gingival recession, while presence of oral piercing was a new factor that was found to be associated with gingival recession.

** Key words:**Gingival recession, prevalence, risk factors, young adults.

## Introduction

Gingival recession [GR] has been defined as an apical shift of the gingival margin over the cementenamel junction [CEJ] and the exposure of the root surface to the oral environment (1). It is a common and undesirable condition that is frequently encountered in dental practices and concerns individuals of all ages throughout the world, describes the condition of periodontal tissue and for this reason it is not considered as a disease itself. Its presence is disturbing for patients due to esthetic, psychological and functional problems, e.g. dentine hypersensitivity, root caries and abrasion, cervical wear, tooth mobility and dental erosion because of the exposure of the root surface to the oral environment (2). Armittage (3) has described forms of GR in the absence of periodontal disease which are known as developmental or acquired deformities and conditions.

The aetiology of GR is multifactorial and is always the result of more than one factor acting together such as anatomical [alveolar bone dehiscence, high muscle attachment, occlusal trauma, frenal pull, thin gingival biotype], inflammatory [destructive periodontal disease, presence of dental plaque and supra/subgingival calculus,inadequate teeth brushing], traumatic factors [vigorous oral hygiene habits, oral piercing] and iatrogenic factors related to reconstructive, conservative, orthodontic, periodontologic or prosthetic treatment (1,2,4,5).

GR can be present in healthy periodontal tissue (2) and appears as wedge shaped lesions on the buccal surface of the teeth, especially in association with malpositioned teeth and hard toothbrush use (6), whereas in individuals with poor oral hygiene it can be present on any tooth surface (7).

Tobacco smoking is considered as one of the main risk factors for the development of destructive forms of periodontal disease and considered as a risk factor associatedwith GR (8).

Previous reports have demonstrated a GR prevalence of 50% or higher percentagesages (1,8-10), whereas Albandar and Kingman (11) and Arowojolu (12) recorded a GR prevalence which ranged from 22.5% to 27.7%, respectively.

A small amount of epidemiological studies has investigated the role of the mentioned factors in the development of GR in young adults in several countries, however a limited amount of similar studies have been carried out in Greece; therefore it is important to collect detailed information, to assess the tendency and epidemiology of this condition, identification the aetiological factors and establish preventive measures.

The aim of the current cross-sectional study was to assess the prevalence and associated factors of GR in an adult population sample in Greece.

## Material and Methods

- Study population

The study sample consisted of 1,430 young adults, 680 males and 750 females aged 18-38 years. Every year in the Greek territory is organized an epidemiological survey in order to be estimated the oral health status of the Greek population regarding several indices of it, by the Greek Health Authorities. The individuals fill in an appropriate questionnaire regarding several aspects of their medicine and dental history and they are being examined by private dentists. The clinical examination and the recording of the oral health status is free of charge in order to establish a representative random sample. As part of the National Oral Health survey the current study was carried out between October to December 2012. It is important to highlight that the issue of the present research was not included in the National Oral Health survey. The participants of the current study were filled in a second questionnaire which included the examined variables and were examined clinically by a private dentist.

- Selection Criteria

The age of the participants ranged from 18 to 38 years old and the minimum number of remaining teeth of each participant should be 20, as more than 12 missing teeth can cause problems with eating, speech, and other basic activities and could lead to overor underestimate the prevalence of GR and the possible associations that are under consideration. None of the participants who had received scaling and root planning or periodontal treatment during the previous six months included in the study.

Third molars and remaining roots excluded from the study, as well.

- Questionnaire 

Before the clinical examination, all participants filled in a questionnaire regarding epidemiological variables [age and gender], smoking status [smoker or non-smokers, occasional smokers excluded from the study],educational level [low:primary and/or secondary educational level and high:college, or university level], income level [0-1,000 € a month and 1,001 € a month and above], use of homecare oral hygiene devices [toothbrush or toothbrush and dental floss], frequency of tooth-brushing daily [one or none times a day and two or more times a day], dental follow up [two times/year and less than one time/year] and presence or absence of previous orthodontic treatment and oral piercing.

The classification of GR, mild, moderate and advanced based on the following criteria determined by Marini *et al.* (13): mild recession:≤ 3.0 mm of root surface exposed tothe oral environment, moderate recession:3.0-4.0 mm of root surface exposed to the oral environment and advanced recession:> 4.0 mm of root surface exposed to the oral environment.

- Clinical examination

The oral clinical examinations were performed in a private dental practice, using a conventional dental unit and illumination, by a qualified in Periodontology dentist. The following indices were recorded in the subsequent order:plaque index [PlI], by Löe (14), gingival index [GI] by Löe and Silness (15) and gingival recession [GR].

Supragingival dental plaque was visualized by the use of a disclosing solution [erythrocin, 3%] and scored as present or absent on all, mesial-buccal-distal-lingual, tooth surfaces. The measurements were performed by means of a William’s manual probe [PCP10-SE, Hu-Friedy Mfg. Co. Inc., Chicago, IL, USA] and they were rounded off to the nearest millimeter; for example a reading of 3.6 mm is recorded as 4.0 mm and a 5.3 mm reading is recorded as 5.0 mm.

If the CEJ of a tooth was destroyed by decay, abrasion, erosion or was covered by acrown or filling, or by dental calculus the estimation of its GR rate was based on the GR rate of the adjacent teeth.

- Ethical considerations

The present study was not an experimental one. The Greek Health Authoritiesapprove and allow only experimental studies under a special permission. Therefore,no permission was required for the performance of the current study.

Subjects who agreed to participate in the present study informed about the evaluation to which they would be submitted and signed an informed consent form. In cases of which the participants had several pathological conditions regarding their oral hygienestatus they were informed about their problems in order to be under a treatment process.

- Reproducibility 

The intraexaminer variance was determined by an additional clinical examination which performed by the same dentist and concerned a sample of 145 [10%] subjects which drawn randomly. After consideration of the code numbers of the double examined participants no differences were recorded between the 1st and the 2nd clinical assessment [Cohen’s Kappa= 0.87].

- Statistical analysis

For each individual the worst values of GR and other periodontal indices at the buccal surfaces of the examined teeth were recorded and then classified based on the mentioned criteria. The classification of GR based on the criteria determined by Marini *et al.* (16) was modified and was coded 0 for absence of GR and/or mild recession [≤ 3.0 mm of root surface exposed to the oral environment] and 1 for moderate and advanced recession [3.0-4.0 mm and > 4.0 mm of root surface exposed to the oral environment, respectively], as dichotomous variables. The classifications of GI and PlI were also modified in order to be used as dichotomous variables.

Statistical analysis of questionnaire items was performed by using a multivariate logistic regression analysis model to identify which variables were best associated with GR. A stepwise selection procedure was used to investigate the influence of possible risk factors on the outcome of GR. A two-step approach was used for this aim. First, bivariate analysis was used to test the relationship between GR and the associated factors. In addition, odds ratios with 95% confidence interval [CI] were used to assess the bivariate relationships among the examined variables. Adjusted odds ratios with 95% CI were assessed as well.

The data analysis was performed using the statistical package of SPSS ver. 17.0 [SPSS Inc., Chicago, IL, USA]. A p value less than 5% [*p*< 0.05] was considered to be statistically significant.

## Results

The total number of the individuals that visited the private practice during the determined period by the Greek Dental Association for their annual dental follow-up was 1,617; however, 1,430 of them were selected to participate in the present study, according to the selection criteria mentioned, 65 did not meet the mentioned criteria and 122 refused to participate in the study, giving a response rate 88.4%.

The mean age of the sample was 28.2 ± 4.2 year old.

The prevalence of GR was overall 63.9%, 68.9% in males and 59.3% in females. No significant difference between males and females regarding GR prevalence was recorded [*p* = 0.121].

The distribution of the sample according to gender and the examined socioeconomic factors, oral health habits, previous orthodontic treatment, smoking status, presence/ absence of oral piercing, supra-gingival calculus, GI, PlI and GR is shown in  Table 1. The results showed that occurrence of GR, was associated with male gender, higher income and educational level, cigarette smoking, previous orthodontic treatment, presence of oral piercing and presence of dental plaque and supra-gingival calculus. The mentioned factors that were associated with the presence of GR, unadjusted and adjusted OR’s and 95% CI are shown in  Table 2.

**Table 1:**
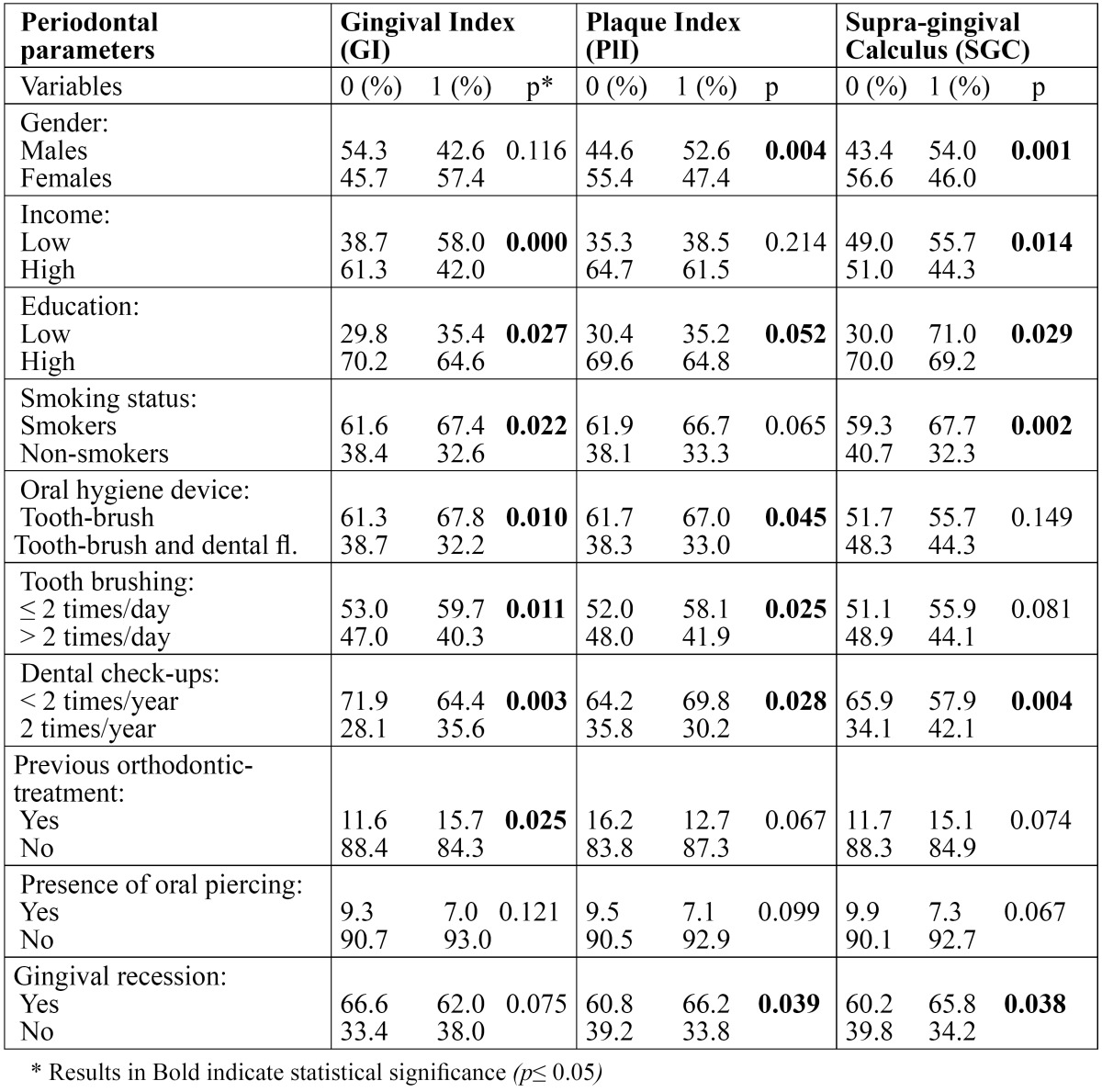
Descriptive characteristics of the study population.

**Table 2:**
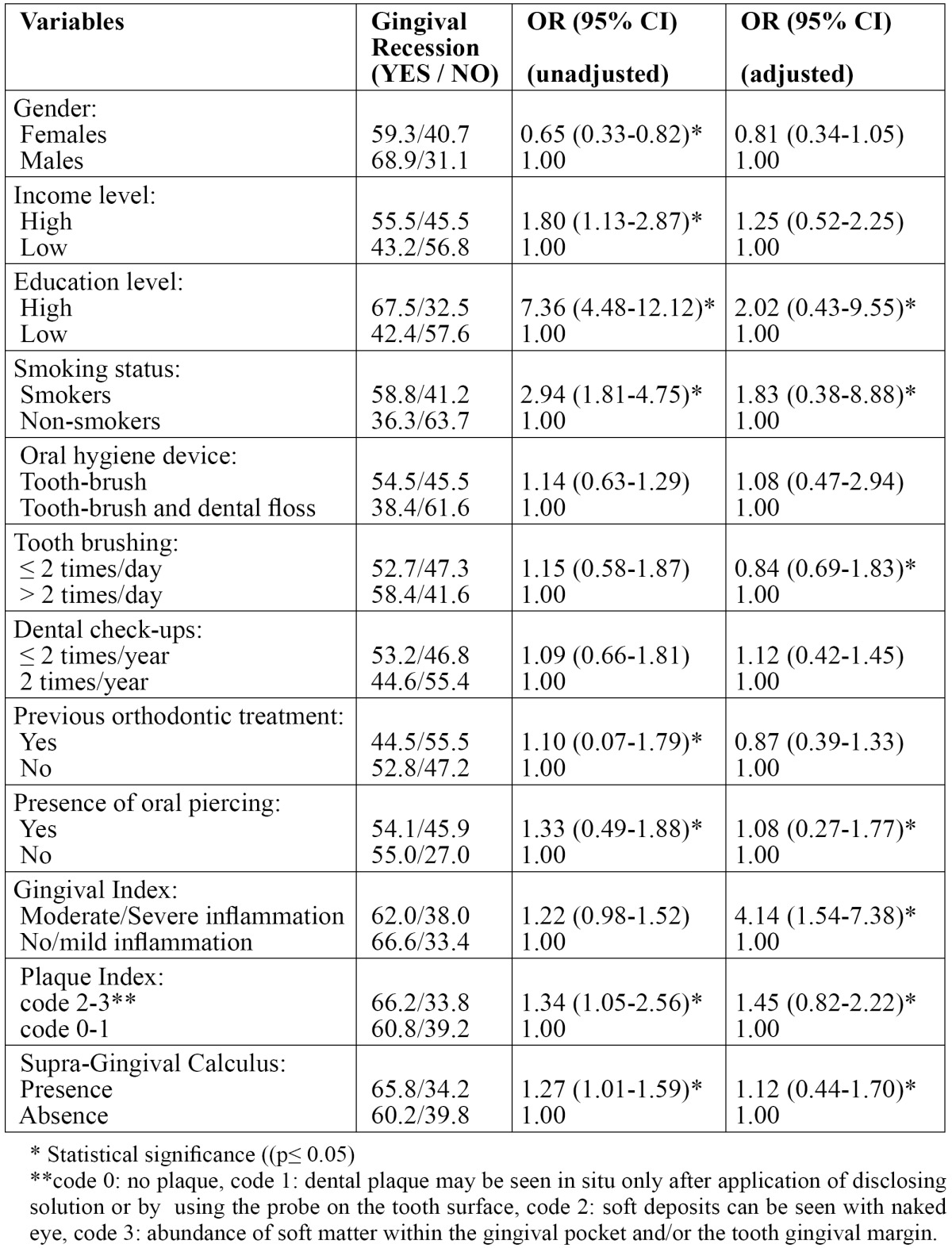
Results after performance of bivariate and multivariate logistic regression analysis with unadjusted and adjusted Odds Ratios and 95% Confidence Interval.

The results after performance of the final model [stepwise method] of the multivariate logistic regression model are presented in  Table 3 and showed that GR was associated with the examined periodontal indices, higher educational level, cigarette smoking frequent tooth brushing and presence of oral piercing. Other conditions such as occlusal trauma, bruxism, thin gingival biotype, previous periodontic treatment that could be considered as factors associated with GR excluded from the study, because of the low rates of the participants that showed the above mentioned conditions.

**Table 3:**
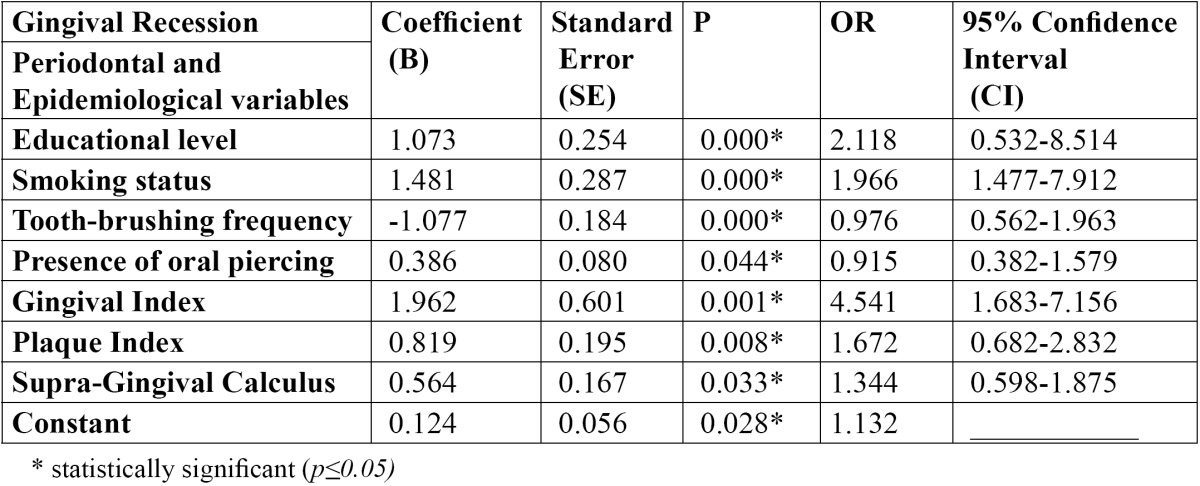
Final multivariate logistic regression model (stepwise model).

## Discussion

As mentioned GR is a common and undesirable condition, concerns individuals of all ages throughout the world and its presence is disturbing for patients due to esthetic, psychological and functional problems. However, according to the literature few studies have investigated the epidemiology and the associated factors of GR, whereas a limited number of similar studies have been carried out in Greece. The current research showed that the prevalence of GR was overall 63.9%, 68.9% in males and 59.3% in females, findings that were in agreement with those from previous report that have recorded that GR prevalence ranged from 50.0% to higher percentages (8-10). Few studies have recorded that GR prevalence ranged from 22.5% (11) to 27.7% (13). Similar findings regarding the distribution of GR by gender were found in previous reports (8-11,16) with higher rates in males than in females. Only in one study (17) was observed that 31.7% of females and 24.3% of males showed GR, finding that could be attributed to the fact that females are more motivated with regard to oral hygiene practices and, thus, brush their teeth more frequently than males (18). The role of dental plaque accumulation and gingival inflammation in the development of GR has been analyzed in previous epidemiological studies in which gingival inflammation was the most frequent precipitating aetiological factor of GR (10,16). Toker and Ozdemir (9) recorded a positive association between high levels of dental and occurrence of GR, finding that was not confirmed by similar studies (10,19). This discrepancy could not dispute the role of dental plaque as a risk factor for GR and could be considered as a random finding.

A weak association was also shown between the presence of supra-gingival calculus and GR, finding that was in accordance with those from previous reports in which the formation of supra/subgingival calculus was found to be one of the most important factors associated with GR (2,8,9,18). The role of dental calculus is important in maintaining and accentuating periodontal disease by keeping dental plaque in close contact with the gingival tissue and creating areas where plaque removal is impossible (9). However, the current view is that dental calculus is not a pathogenic factor in periodontal diseases, but enhances gingival inflammation by promoting and retaining new plaque (20). In another report in Greece no association was recorded between the examined variables (10).

The analysis of the results showed a weak but negative association between tooth-brushing frequency and occu-rrence of GR as 58.4% of the individuals that brushed their teeth two or more times/day showed GR. This finding was in accordance with those from previous reports in which GR was associated with vigorous, frequent, forceful and excessive use of medium hardness or hard toothbrushes in an horizontal direction (17,21) and generally mechanical trauma from tooth brushing (9,12). These observations may be explain the development of GR in individuals with a good standard of oral hygiene. In contrast to the above observations, other researches recorded no significant differences between GR and toothbrush type and frequency of tooth-brushing (18,22). Rajapakse *et al.*(23) in a systematic review recorded that out of 17 studies only 2 concluded that there appeared to be no relationship between tooth brushing frequency and GR while 8 studies reported a positive association between tooth brushing frequency and GR. The mentioned observations confirm that exists a need to educate the patients to use proper tooth-brushing methods and other available means for dental plaque control (dental floss, inter-dental brushes, oral solutions]. The application of effective oral hygiene habits results in less plaque accumulation, less calculus formation and less periodontal disease and GR.

Tobacco smoking was associated with the occurrence of GR in the present and previous studies. These studies showed that tobacco smoking was regarded as one of the main risk factors for development of destructive forms of periodontal disease (4,10,16). They also suggested that the combination of smoking and supragingival calculus was associated with localized and generalized GR (8,9,12,18) and that smoking may be a risk factor for GR in adults with minimal periodontal destruction (24). However, in a report by Muller *et al.* (25) smoking status was not identified as a risk factor for the development of GR, while similar studies suggested a negative impact on GR and periodontal health from tobacco smoking (26,27).

Despite the mentioned conflicting observations there is an established literature on the relation ship between smoking and periodontal disease pathogenesis (28), development and progression of periodontal disease, destructive periodontal disease, alveolar bone loss and poor response to periodontal therapy, although the mechanisms of its negative influence are not well understood (29).

The influence of educational level on occurrence of GR was evident. This observation agrees with previous reports suggesting that educational level was an important contributor to buccal GR (30) whereas in two reports (8,10) no association was recorded between the examined variables. The presence of such an association could be attributed to the fact that more educated individuals have realized the value and importance of preventive dentistry and oral hygiene, have a good standard of oral hygiene, use the available means for dental plaque control and follow a regular dental follow-up.

Oral piercing is another cultural-causative factor of GR (4,19). Despite the fact that Greek society is considered as a conservative one, nearly 8.0% of the individuals in the current report applied that cultural factor in their mouth, while a weak and positive association between presence of oral piercing and occurrence of GR was recorded. The differences that have observed among the available previous studies regarding the association between GR and the examined variables could be attributed to several factors such as the heterogeneous population samples, the different study designs for collecting data regarding the used tools, questionnaire, x-rays or health questionnaires, the origin of the participants in order to establish a representative sample, etc. The sample of the present study concerned subjects who visited a private dental practice for its annual dental follow-up and could be considered as a representative and a random one. In addition it is important to highlight that the a etiology of GR is multi factorial and its occurrence is always the result of more than one factor acting together. As mentioned few studies have investigated the combination of possible risk factors of GR. These studies have shown associations and indicative risk factors but they have not identified the aetiological factors, because for this aim prospective studies could be necessary. Other studies are still necessary to explain the etiology of GR, focusing in the biological, chemical and behavioral factors involved in order to implement adequate preventive policies.

There are probably many more implicating factors other than the ones already stated in the current study in the initiation of GR that may not have been considered in the present study.
